# Supplementation of laying hens’ feed with Schizochytrium powder and its effect on physical and chemical properties of eggs

**DOI:** 10.1186/s12917-024-04424-x

**Published:** 2024-12-06

**Authors:** Ifra kiran, Huma Umbreen, Mahr un Nisa, Fahad Al-Asmari, Eliasse Zongo

**Affiliations:** 1 Department of Nutritional Sciences, Government College University Faisalabad, Faisalabad, Punjab 38000 Pakistan; 2 Department of Food and Nutrition Sciences, College of Agricultural and Food Sciences, King Faisal University, Al-Ahsa, 31982 Saudi Arabia; 3 Laboratory of Research and Teaching in Animal Health and Biotechnology, Nazi Boni University, Bobo-Dioulasso, Dindéresso, 1091 Burkina Faso

**Keywords:** Microalga, DHA, *Schizochytrium limacinum*, N-3 PUFA, Fortification of eggs

## Abstract

Biofortification enhances dietary quality and nutritional value using traditional marine microalga species, particularly docosahexaenoic acid (DHA), which is an essential n-3 fatty acid for human health. Eggs are natural fortified candidates. This study aimed to investigate the effect of dietary Schizochytrium powder on egg productivity, egg nutritional content, and fatty acid levels in laying hens. Hy-Line Brown laying hens (*n* = 150) were uniformly assigned to five groups for 52 days. The control group received no supplements, whereas the other four experimental groups were provided with varied amounts of schizochytrium powder. The experimental feed contained isonitrogenous (16.60%) and isocaloric metabolizable energy, 36.69 MJ/kg components. No significant differences were observed (*p* > 0.05) on different parameters such as average daily feed intake, feed conversion ratio, egg weight, and shell thickness. Enriching eggs significantly increased α-linolenic acid (ALA), eicosapentaenoic acid (EPA), and DHA levels (*p* < 0.001), while decreasing the n-6/-3 ratio in a dose-dependent manner, ensuring high quality and biological efficacy. A plateau point was maintained throughout the study period. In the first two weeks, increased DHA levels were observed in egg yolks when microalgae concentrations were elevated by 1%. The study found that powdered *Schizochytrium limacinum* served as a substitute for DHA in enhancing eggs with n-3 fatty acids.

## Introduction

Growing health awareness among consumers is driving the demand for functional foods with essential nutrients, leading to a market for products enriched with bioactive compounds [1]. Among these compounds, polyunsaturated fatty acids (PUFAs) including α-linolenic acid (ALA), eicosapentaenoic acid (EPA), docosapentaenoic acid (DPA), and docosahexaenoic acid (DHA) are renowned for their positive effects on development and growth [2]. These are vital components of biological membranes, brain and are modulators of enzymes and immune functions [3, 4]. The body can convert ALA to EPA and DHA, however higher omega 6 to omega 3 ratio limits DHA synthesis [5]. Furthermore, the western diet has disrupted the optimal balance between n-6 and n-3 PUFA, and shifted it to a disproportionate ratio i.e. 15–20:1 [6]. According to European Food Safety Authority (EFSA) the daily recommended intake of EPA and DHA ranges from 140 to 600 mg/day for adult, which is not followed by most western communities [7].

A potential solution lies in natural additive-free DHA-enriched foods to address this gap. The push to incorporate EPA/DHA into diets has generated interest in enhancing food products, propelling continuous research and development efforts [8]. From a nutritional perspective, eggs are prime candidates for dietary enhancement, and various oils such as linseed, soybean, sunflower, and fish oils are used to produce specialized eggs. Typically, eggs contain a significant amount of n-6 PUFA but lack a proportionate amount of n-3 PUFA [9]. Eggs are important as our direct diet and as ingredients in various dishes, making them ideal candidate for enrichment with n-3-PUFA. Regular consumption of DHA-enriched eggs addresses human dietary deficiencies, lowers the risk of fatal heart disease, regulates brain function and may also encourage consistent consumption [10].

Fish oil, a conventional means of introducing DHA to enhance egg production, is susceptible to oxidation, leading to undesirable flavors. The cost and diminishing fish resources further raise concerns regarding sustainability. Marine microalgae (seaweeds which are originally responsible for n-3 PUFA in marine food chains, have emerged as an alternative to fish oil for enriching eggs with DHA. Furthermore, the inclusion of microalgal DHA into egg yolk introduces antioxidants through carotenoids, gradually enhancing the stability of yolk lipids by safeguarding vulnerable PUFA [11]. Numerous varieties of microalgae, including the DHA-abundant *Schizochytrium limacinum*, are available for enrichment of different foods and can be a justified substitute for fish oil in the process of enriching table eggs with n-3 fatty acids.

Our research aimed to investigate the influence of incorporating the microalgae *Schizochytrium limacinum* into laying hens feed as an additive. This study specifically focused on assessing their effects on the composition of fatty acids, with a special emphasis on the content of n-3 PUFA. The present study also focused on the plateau point of the diet at which the concentration of supplementation reaches a maximum in eggs and is maintained at an optimal level.

## Materials and methods

### Animal sample collection and their management

A total of 150 Hy-Line Brown hens, aged 30 weeks, were housed within the poultry experimental facility situated at the Control Shed of the Pelwan Poultry Farm. 71 JB, Sarlian Jhang Road, Faisalabad, Pakistan. This housing was maintained under standardized conditions involving a 12-hour day/night cycle, a consistent environmental temperature of 25 °C, and appropriate ventilation. Water and food were provided ad libitum throughout the trial period.

### Ethical considerations

All steps of this experiment were used in accord with Internationally Accepted Guidelines for Animal Research as Prescribed by the Declaration of Helsinki. All animal procedures were approved by the Animal Ethics Committee of the Government College University, Faisalabad, Pakistan. Members of the Institutional Review Board, Government College University, Faisalabad, checked the research plan and issued a letter signed by the members of the Review Board (Ref No. GCUF/ERC/309).

### Experimental design

The experiment was conducted over 52 d. The study was designed with five groups: one control treatment was without algae (Schizochytrium powder), and the other four groups were treated with different algae treatments, as shown in Table 1. There were total 150 hens which have been divided in 5 groups (total 30 in each group) with 3 replicates (10 hens in each replicate) in each group. The hens did not receive any vaccines or drugs during the experimental period. *Schizochytrium limacinum* powder was kindly donated by Alltech distributors, Pakistan.

**Table 1:** Experimental design for enrichment of eggs with *Schizochytrium Limacinum*

Groups	Treatment plan
Group 1	Control group with normal feed
Group 2	Normal feed with 0.25%, Schizochytrium algae powder
Group 3	Normal feed with 0.50%, Schizochytrium algae powder
Group 4	Normal feed with 0.75%, Schizochytrium algae powder
Group 5	Normal feed with 1%, Schizochytrium algae powder

The hens were given normal feed for two weeks to help them adapt to both their feed and the surrounding environment. This period also allowed for the collection of initial data (week 0). The hens were randomly allocated to four different feed treatments. The compositions of the experimental and control (normal) feeds are given in Table 2.

**Table 2:** Composition and nutrient levels of the experimental feeds

Grouping	Control	0.25%	0.50%	0.75%	1%
Ingredients	Dietary composition of treatment Groups (%)				
Soybean meal	23.40	22.81	19.20	18.11	17.40
Soybean oil	0.05	0.04	0.03	0.02	0.01
Maize	41.9	41.9	41.9	41.9	41.9
Limestone	9	9	9	9	9
Cysteine	1	1.5	1.7	1.8	1.9
Yeast	5.0	5.0	5.0	5.0	5.0
Alfalfa	1.67	1.67	1.67	1.67	1.67
Sodium Chloride	0.30	0.30	0.30	0.30	0.30
Methionine	0.33	0.35	0.36	0.37	0.38
Lysine	0.89	0.89	0.89	0.89	0.89
Calcium	3.37	3.37	3.37	3.37	3.37
Premix^2^	0.68	0.68	0.68	0.68	0.68
Methionine	0.15	0.23	0.23	0.23	0.23
Wheat	10.1	10.1	10.1	10.1	10.1
Zeolite powder	0.10	0.10	0.10	0.10	0.10
Choline chloride	0.9	0.9	0.9	0.9	0.9
Sugar	0.12	0.12	0.12	0.12	0.12
Lysophosphatidylcholine	0.1	0.1	0.1	0.1	0.1
Starch	0.03	0.03	0.03	0.03	0.03
Available phosphorus	0.33	0.33	0.33	0.33	0.33
Potassium	0.91	0.91	0.91	0.91	0.91
Nutrient level	100%				
**Chemical Analyzed Nutrients Level (%)**					
Ash	19.3	14.4	13.4	11.8	15.4
Crude fibers	34	31	31	33	32
Crude Fat	77	76	79	80	77
Moisture	82	82	85	86	84
Crude protein	16.81	16.81	16.81	16.81	16.81
Methionine + cysteine	0.65	0.65	0.65	0.65	0.65
**Metabolizable energy in Megajoules per kilogram (MJ ME kg-1)**					
Total Energy	36.33	36.89	37.11	37.547	36.696

Premix [2] contains both minerals and vitamins (vitamin A, vitamin E, vitamin K3, vitamin B1, vitamin B2, vitamin B3, vitamin B6, vitamin B12, vitamin PP, folic acid, biotin, choline, Fe, Cu, Sn, Mn, I, Se, and Zn. Co, ethoxyquin)

### Physical evaluation

#### Feed conversion ratio

The feeding trial was conducted at 0, 14, 28, and 52 days for the analyses. On these trial days, three from each replicate of a group were collected in the afternoon for assessment and their mean values were calculated for Egg mass. The feed conversion ratio (FCR), daily feed intake (DFI) and egg mass were calculated as follows:

$$\:Feed\:Conversion\:Ratio=\frac{Daily\:Feed\:intake\:\left(g\right)}{Egg\:mass\:\left(g\right)}$$

Daily Feed intake (DFI) was calculated using the following equation:

$$\:Daily\:Feed\:intake\:\left(g\right)=\frac{Feed\:consumption\left(g\right)}{Hen\:number\:\times\:Days}$$

Similarly egg mass was calculated using following formula:

$$\:Egg\:Mass\:\left(g\right)=Egg\:production\:\left(\%\right)\times\:Mean\:egg\:weight$$

#### Egg yolk color

The DSM yolk color fan, manufactured by DSM Nutritional Products Ltd, Switzerland, was used to measure egg yolk color using a 15-point color scale ranging from 15 (dark orange) to 1 (light pale) using the method of Adrizal et al. [12].

#### Thickness of the eggshell

The thickness of the eggshell, after the removal of the egg content and egg membranes, was measured with a micrometer at the pole and equator of the egg using an eggshell thickness gauge [13]. The measurements were taken for all the collected eggs of a group and the value were presented as average.

#### Heights of the yolk and albumen

A tripod digital micrometer was used to measure the heights of the yolk and albumen. Albumen height was measured midway between the yolk and the thickened albumen. Albumen thickness and egg weight were used to calculate the Haugh unit (HU) for egg quality using the following formula:

$$\:HU=100\:\text{l}\text{o}\text{g}[H+7.57--1.7\:W-37]$$

#### pH of the egg

To check the pH of the egg, albumin and yolk were analyzed separately, and an Accumet 950 pH/ion meter was used to evaluate pH [14].

### Biochemical evaluation of enriched eggs

#### Determination of crude protein

The traditional standard procedure for determining the protein level is Kjeldahl analysis (AOAC 988.05), which measures total nitrogen. This approach was employed to calculate the crude protein concentration in the eggs [15].

Crude protein was calculated as,

$$\:Crude\:Protien\:\left(\%\right)=\:6.25\times\:\%\:Nitrogen$$

#### Determination of crude fat

For determination of fat, moisture-free samples were analyzed in a Soxtech system using the methods of Carrillo et al. [16] and the fat percentage of the sample was calculated as follows:

$$\:\text{Crude\:fat}\text{(}\text{\:\%}\text{)}\text{\:=\:}\frac{\text{W}\text{eight\:of\:ether\:extract}}{\text{W}\text{eight\:of\:original\:sample}}\times100$$

#### Determination of ash content

The ash contents of the samples were evaluated under AOAC 942.05 settings using a muffle furnace [17]. The crude ash contents of the samples were calculated using the following formula:

$$\:Crude\:Ash\:\left(\%\right)=\:\frac{(R-T)}{(W-T)}\times\:100$$

where W is the weight of the crucible and test portion, R is the weight of the crucible and residue, and T is the (void) weight of the crucible.

#### Gas chromatography

Gas chromatography was used to evaluate fatty acid methyl esters according to the method described by Feng et al. [18]. The sample was ether extracted using the Soxtech method, and pyrogallic acid was then added to prevent oxidative decomposition of the fatty acids throughout the evaluation. Methylation of the fat resulted in fatty acid methyl esters (FAME) when boron trifluoride (BF_3_) in methanol was employed. A gas chromatograph fitted with an automatic sampler, a hydrogen flame ionizing sensor set at 250 °C, a 60 m long vertical column with a diameter inside of 0.25 mm and an outer density of the film of 0.25 μm, was used for accurate measurement of FAME [19].

### Statistical analysis

The results were evaluated using two-way ANOVA followed by a post hoc least significant difference (LSD) test for multiple comparison, using IBM SPSS Statistics 21 software. The data are presented as means and cumulative SEM between the treatment groups and experimental days. The treatment means were considered statistically significant at *p* ≤ 0.05.

## Results

### Analysis of egg and its composition

On day 0, all the experimental groups had equivalent egg weights. Egg weights increased in all groups during the duration of the trial period, irrespective of the group, but there was no significant difference (*p* = 0.9013) between the groups in accordance with the treatment over the period from 0 to 52 days. Similarly, eggshell thickness was constant across all groups throughout the study period and there were no significant differences (*p* ≥ 0.05) between the control and treatment groups (Table 3). However, a significant difference (*p* ≤ 0.05) was observed in yolk color (Table 3) between the different treatment groups over the study duration from days 0 to 52. Compared with the control group, the color of the egg yolk got darker in hue in all treatment groups. The 1% treatment group improved greatly in yolk shade (7.66 ± 0.04) on day 28, which was maintained till day 52 showing no statistical difference during this time. Conversely, no significant difference was observed in yolk color of the control group throughout the study period.

Albumin height, an indicator of egg white thickness, was spiked with the duration of different treatments. However, no significant difference was observed between the different supplementation groups during the study period from 0 to 52nd day (Table 3). On day 52, the 1.00% treatment group exhibited an increased albumin height (8.03 ± 0.03 mm), although, not significant different overall. The hue, consistency, texture, and quality of the egg yolk and albumin are all dependent on the stabilized pH levels. The results for egg albumin and yolk pH are shown in Table 3. Albumin pH maintained mostly uniform, between 7.62 ± 0.36 to 8.44 ± 0.17 and no significant difference (*p* = 0.3802) was observed between different treatment groups over the study duration. Furthermore, the yolk pH showed a significant difference between the groups and the study period from day 0 to 52. The pH of the yolk ranged from 5.80 ± 0.01 to 6.12 ± 0.03 for different groups, and the highest was observed for group consuming 0.75% Schizochytrium powder on day 52 and lowest on day 0 of 1% treatment group, which was not significantly different from day 0 of the other groups also.

Moreover, daily feed intake (DFI) was not found to be significantly different among the different groups throughout the study duration (Table 3); however, the feed conversion ratio (FCR) demonstrated a significant difference between the treatments during the study period from day 0 to 52, where the results showed a mixed trend. The lowest FCR was observed day 0 in control group, which was not significantly different from day 0 of treatment groups as well as day 52 and day 28 of 1% and 0.25% treatment groups, respectively; similar trend was also observed in 0.5% and 0.75% treatment groups. In all groups, irrespective of treatment, Haugh unit (HU) tended to be constant throughout the study duration (Table 3) and the results showed no statistically significant (*p* = 0.7341) variations in HU among the tested groups.

The chemical composition of the eggs also showed mixed trends in the levels of crude protein, fat, and ash (Table 3). Crude protein showed no significant difference between the different treatment groups throughout the study duration (*p* = 0.612), whereas crude fat was significantly different between the groups and treatment period (0–52 days). The highest fat content was observed in the 1.0% treatment group on day 28 (28.97 ± 0.19%), which was maintained until day 52 by this group. A similar trend was observed in the other treatment groups. Furthermore, the ash content of all the treatment groups differed significantly (*p* ≤ 0.05) during the study period. The highest ash content was observed in 0.25% treatment group on day 14, which was maintained again on day 52 after a fall on day 28. similar trend was also observed for 1.0% and 0.5% treatment groups, whereas 0.75% treatment group showed an increasing trend until day 28 afterwards a significant reduction was observed on day 52.

**Table 3:** Values (Mean ± SEM) for physical and chemical parameters of laying eggs

Parameters	Treatment groups					
	Control	0.25%	0.50%	0.75%	1%	SEM
Egg Weight (g) 0 day 14 days 28 days 52 days	56.26 ± 0.21 56.26 ± 0.21 57.55 ± 0.26 57.96 ± 0.37	56.27 ± 0.26 58.47 ± 0.26 59.42 ± 0.36 59.46 ± 0.23	56.26 ± 0.24 56.54 ± 0.24 59.33 ± 0.32 59.52 ± 0.23	56.25 ± 0.22 56.70 ± 0.23 59.31 ± 0.28 59.38 ± 0.28	56.27 ± 0.21 56.95 ± 0.22 58.37 ± 0.26 59.53 ± 0.33	0.8513
**P-value Groups**	0.06					
**P-value Days**	0.002					
P-value Groups*Days	0.901					
**Egg Shell (mm)** 0 day 14 days 28 days 52 days	0.35 ± 0.09 0.35 ± 0.04 0.37 ± 0.09 0.35 ± 0.07	0.35 ± 0.09 0.35 ± 0.07 0.35 ± 0.08 0.37 ± 0.09	0.35 ± 0.04 0.34 ± 0.05 0.36 ± 0.04 0.36 ± 0.09	0.37 ± 0.08 0.37 ± 0.08 0.35 ± 0.03 0.36 ± 0.04	0.36 ± 0.04 0.36 ± 0.08 0.36 ± 0.06 0.36 ± 0.04	0.8255
**P-value Groups**	0.928					
**P-value Days**	0.120					
P-value Groups*Days	0.194					
**Egg Yolk Color** 0 day 14 days 28 days 52 days	5.00 ± 0.07^cd^ 5.00 ± 0.05^cd^ 5.36 ± 0.04^cd^ 5.66 ± 0.06^bcd^	5.00 ± 0.02^cd^ 5.66 ± 0.03^bcd^ 5.66 ± 0.03^bcd^ 6.46 ± 0.02^abc^	5.01 ± 0.04^cd^ 5.78 ± 0.03^bcd^ 6.33 ± 0.02^abc^ 6.30 ± 0.02^abc^	5.00 ± 0.03^cd^ 5.90 ± 0.02^bcd^ 6.89 ± 0.03^b^ 6.76 ± 0.04^b^	5.00 ± 0.03^cd^ 7.25 ± 0.02^ab^ 7.66 ± 0.04^a^ 7.66 ± 0.05^a^	0.6777
**P-value Groups**	0.859					
**P-value Days**	0.090					
P-value Groups*Days	0.042					
**Albumen Height (mm)** 0 day 14 days 28 days 52 days	6.40 ± 0.07 6.40 ± 0.06 6.97 ± 0.05 6.96 ± 0.08	6.48 ± 0.08 6.59 ± 0.07 7.12 ± 0.08 7.31 ± 0.06	6.41 ± 0.06 6.48 ± 0.07 7.67 ± 0.04 7.80 ± 0.09	6.40 ± 0.05 6.78 ± 0.05 7.65 ± 0.06 7.36 ± 0.04	6.41 ± 0.05 6.94 ± 0.07 7.33 ± 0.04 8.03 ± 0.03	0.3319
**P-value Groups**	0.107					
**P-value Days**	0.001					
P-value Groups*Days	0.090					
**Albumen pH** 0 day 14 days 28 days 52 days	7.75 ± 0.12 7.74 ± 0.23 7.75 ± 0.29 7.96 ± 0.47	7.75 ± 0.12 8.01 ± 0.27 8.44 ± 0.17 7.65 ± 0.12	7.75 ± 0.17 8.19 ± 0.19 7.86 ± 0.23 7.84 ± 0.31	7.73 ± 0.43 8.10 ± 0.36 8.21 ± 0.33 7.95 ± 0.29	7.74 ± 0.52 7.87 ± 0.27 7.73 ± 0.39 7.62 ± 0.36	0.2041
**P-value Groups**	0.218					
**P-value Days**	0.060					
P-value Groups*Days	0.380					
**Yolk pH** 0 day 14 days 28 days 52 days	5.81 ± 0.02^d^ 5.81 ± 0.03^d^ 5.90 ± 0.02^c^ 5.89 ± 0.03^c^	5.82 ± 0.03^d^ 5.81 ± 0.06^d^ 5.94 ± 0.01^bc^ 5.97 ± 0.01^b^	5.81 ± 0.04^d^ 5.89 ± 0.02^c^ 5.89 ± 0.02^c^ 6.03 ± 0.05^ab^	5.87 ± 0.04^c^ 5.81 ± 0.05^d^ 6.09 ± 0.04^ab^ 6.12 ± 0.03^a^	5.80 ± 0.01^d^ 5.89 ± 0.02^c^ 6.00 ± 0.02^ab^ 5.99 ± 0.02^ab^	0.0395
**P-value Groups**	0.036					
**P-value Days**	0.000					
P-value Groups*Days	0.000					
**DFI (g/Hen)** 0 day 14 days 28 days 52 days	103.67 ± 5.21 103.67 ± 4.13 106.67 ± 4.11 102.0 ± 6.22	102.67 ± 3.12 112.0 ± 2.27 110.6 ± 2.26 116.97 ± 4.16	102.7 ± 3.19 107.33 ± 3.11 111.33 ± 4.67 104.33 ± 3.27	103.67 ± 2.46 110.67 ± 2.66 113.0 ± 2.35 115.67 ± 3.71	104.67 ± 6.21 107.0 ± 6.67 111.0 ± 6.71 109.0 ± 3.11	4.6389
**P-value Groups**	0.075					
**P-value Days**	0.555					
P-value Groups*Days	0.198					
**FCR** 0 day 14 days 28 days 52 days	1.19 ± 0.15^f^ 1.40 ± 0.17^abcd^ 1.62 ± 0.23^abcd^ 1.52 ± 0.17^def^	1.20 ± 0.16^f^ 2.14 ± 0.13^ab^ 1.19 ± 0.11^f^ 2.17 ± 0.13^ab^	1.19 ± 0.14^f^ 2.08 ± 0.20^abc^ 1.78 ± 0.21^bcd^ 1.93 ± 0.15^abcd^	1.18 ± 0.15^f^ 2.28 ± 0.18^a^ 2.21 ± 0.19^a^ 1.69 ± 0.12^cde^	1.36 ± 0.27^ef^ 2.07 ± 0.21^abc^ 1.95 ± 0.22^abc^ 1.36 ± 0.19^ef^	0.2020
**P-value Groups**	0.003					
**P-value Days**	0.112					
P-value Groups*Days	0.000					
**Haugh Unit (HU)** 0 day 14 days 28 days 52 days	78.63 ± 4.68 78.15 ± 2.18 78.03 ± 2.82 85.48 ± 3.40	78.03 ± 3.33 79.54 ± 3.19 79.51 ± 3.81 79.12 ± 4.07	78.03 ± 2.55 78.09 ± 2.41 83.09 ± 3.92 84.41 ± 3.12	78.9 ± 1.42 79.43 ± 1.67 78.96 ± 1.22 83.91 ± 2.70	79.33 ± 3.66 78.84 ± 3.27 85.40 ± 3.16 84.81 ± 3.68	2.9168
**P-value Groups**	0.010					
**P-value Days**	0.656					
P-value Groups*Days	0.751					
**Crude Protein (%)** 0 day 14 days 28 days 52 days	16.40 ± 0.09 16.41 ± 0.09 16.83 ± 0.15 16.86 ± 0.13	16.41 ± 0.08 16.70 ± 0.16 16.98 ± 0.07 17.02 ± 0.19	16.42 ± 0.13 16.70 ± 0.13 17.18 ± 0.15 17.01 ± 0.09	16.42 ± 0.17 16.62 ± 0.19 16.90 ± 0.12 16.64 ± 0.09	16.41 ± 0.06 16.55 ± 0.03 16.88 ± 0.07 16.70 ± 0.07	0.1051
**P-value Groups**	0.000					
**P-value Days**	0.007					
P-value Groups*Days	0.547					
**Crude Fat (%)** 0 day 14 days 28 days 52 days	27.65 ± 0.17^d^ 27.91 ± 0.19^c^ 27.93 ± 0.18^bc^ 27.59 ± 0.19^d^	27.66 ± 0.21^d^ 27.81 ± 0.23^c^ 28.01 ± 0.24^bc^ 28.72 ± 0.24^ab^	27.64 ± 0.18^d^ 27.94 ± 0.19^bc^ 28.09 ± 0.19^bc^ 28.04 ± 0.18^bc^	27.66 ± 0.24^d^ 27.97 ± 0.22^bcd^ 28.77 ± 0.22^ab^ 28.81 ± 0.25^ab^	27.64 ± 0.21^d^ 28.66 ± 0.23^b^ 28.97 ± 0.19^a^ 28.93 ± 0.21^a^	0.1932
**P-value Groups**	0.001					
**P-value Days**	0.003					
P-value Groups*Days	0.001					
**Ash (%)** 0 day 14 days 28 days 52 days	2.27 ± 0.24^e^ 2.50 ± 0.21^de^ 2.75 ± 0.19^bcde^ 2.51 ± 0.22^de^	2.27 ± 0.17^e^ 3.24 ± 0.21^a^ 2.67 ± 0.16^e^ 3.24 ± 0.14^a^	2.27 ± 0.17^e^ 3.21 ± 0.21^abc^ 2.68 ± 0.25^bcde^ 3.19 ± 0.25^abc^	2.26 ± 0.19^e^ 3.13 ± 0.22^ab^ 3.20 ± 0.21^abc^ 2.73 ± 0.24^bcde^	2.28 ± 0.23^e^ 3.15 ± 0.18^ab^ 3.02 ± 0.18^abcd^ 3.13 ± 0.17^ab^	0.2088
**P-value Groups**	0.000					
**P-value Days**	0.954					
P-value Groups*Days	0.001					

Different dosages of Schizochytrium powder (0.25%, 0.50%, 0.75% and 1%), DFI = Daily Feed Intake; FCR = Feed conversion ratio; SEM is the standard error of means between groups*days

^a−h^ Different letters represent statistical differences groups*days within a parameter at (*p* ≤ 0.05)

### Composition of fatty acids in enriched eggs yolks

On day 0, the levels of docosahexaenoic acid (DHA) found in egg yolks were comparable within groups (Table 4). After 14 days of supplementation, all groups had significantly different egg DHA content (*p* = 0.000). The plateau analysis estimated the duration of all feeds that need to be fed to the egg DHA content to attain a constant level of fortification. No plateau was observed for the un-supplemented control group. At day 14, the 1.0% Schizochytrium group had the greatest DHA concentration in the yolks of eggs, hitting 4.18 ± 0.08 g/100 g yolk. Hens treated with 0.75% and 0.50% Schizochytrium showed a delayed but continuous rise in DHA levels, culminating at 3.14 ± 0.07 g/100 g and 2.03 ± 0.04 g/100 g yolk respectively on day 28. Moreover, DHA levels reached at 1.04 ± 0.05 g/100 g yolk at day 52, in the 0.25% Schizochytrium treatment group, demonstrating a slow fortification over an extended period with a lower dose.

Furthermore, the hens provided with treatment of 1.0% Schizochytrium powder had a significant increase in α-Linolenic acid (ALA) concentration in its yolks, hitting 0.74 ± 0.01 g/100 g yolk around day 14, thereafter the level of ALA reached its plateau point (Table 4). Constant increases in enrichment were found with an increase of the duration of feeding from day 0 until day 14, which was almost maintained afterwards till the end of the experiment (0.70 ± 0.01 g/100 g of yolk). The 0.75% and 0.50% Schizochytrium treatment groups showed a prolonged enrichment phase with a steady increase in ALA concentrations until day 28, after which a constant level was observed through the day 52. Hens treated with 0.25% Schizochytrium had a preliminary ALA enrichment, reaching 0.61 ± 0.02 g/100 g yolk at day 52, demonstrating a progressive and persistent rise throughout the treatment period.

Similarly, hens provided with 1% Schizochytrium powder laid the eggs with enhanced Ecosapentaenoic acid (EPA) content reaching 0.27 ± 0.01 g/100 g yolk at day 14 (Table 4). The EPA contents increased significantly, more slowly but consistently in the 0.75% Schizochytrium group, reaching at 0.26 ± 0.02 g/100 g yolk on day 28, while the hens in the 0.50% Schizochytrium group showed a similar pattern on day 52. However, the EPA content gradually increased in the 0.25% Schizochytrium group reaching the highest at day 28 but then again a decline was observed at day 52 (Table 4).

In the present study, the addition of Schizochytrium to basal diets improved the nutritional value of eggs by increasing both n-3 and n-6 polyunsaturated fatty acids (PUFAs) (Table 4). All groups receiving treatments had similar amounts of n3PUFA and n6PUFA at the start (day 0), with no significant variations. Nevertheless, after 14 days, n3PUFA levels improved significantly among all treatment groups, with the 1% dose regularly producing the greatest omega-3 enrichment at 4.77 ± 0.17 g/100 g yolk (*p* ≤ 0.05), highlighting the efficacy of this dietary supplementation amount. However, no significant difference (*p* = 0.1349) was observed in n6PUFA levels between the groups throughout the study period of 52 days. Additionally, the n-6/3PUFA ratio, a crucial measure of food quality, was found to be the lowest in the 1% group (*p* ≤ 0.05) at day 14 which was maintained till the end of the study period i.e. day 52. Furthermore, almost similar trend was observed for 0.25, 0.5 and 0.75% dose of algae powder which showed the lowest ratio at day 28 that maintained till end of study period.

**Table 4:** Fatty acid components (g/100 g ± SEM) in eggs of different groups in different days

Component	Treatment groups					
	Control	0.25%	0.50%	0.75%	1%	SEM
DHA 0 day 14 days 28 days 52 days	0.50 ± 0.03^i^ 0.60 ± 0.02^i^ 0.52 ± 0.04^i^ 0.50 ± 0.04^i^	0.51 ± 0.02^i^ 0.73 ± 0.08^h^ 0.98 ± 0.05^g^ 1.04 ± 0.05^g^	0.50 ± 0.03^i^ 1.51 ± 0.03^f^ 2.03 ± 0.04^e^ 1.99 ± 0.09^e^	0.52 ± 0.04^i^ 2.27 ± 0.09^d^ 3.14 ± 0.07^c^ 3.13 ± 0.07^c^	0.50 ± 0.02^i^ 4.18 ± 0.08^a^ 4.02 ± 0.08^b^ 4.10 ± 0.09^ab^	0.0499
**P-value Groups**	0.000					
**P-value Days**	0.000					
P-value Groups*Days	0.000					
**ALA** 0 day 14 days 28 days 52 days	0.44 ± 0.01^gh^ 0.45 ± 0.01^g^ 0.42 ± 0.02^hi^ 0.40 ± 0.02^i^	0.44 ± 0.02^gh^ 0.41 ± 0.00^i^ 0.48 ± 0.01^f^ 0.61 ± 0.02^cd^	0.44 ± 0.03^gh^ 0.44 ± 0.02^gh^ 0.59 ± 0.01^de^ 0.58 ± 0.01^e^	0.43 ± 0.00^gh^ 0.45 ± 0.01^g^ 0.63 ± 0.03^c^ 0.62 ± 0.00^c^	0.42 ± 0.01^hi^ 0.74 ± 0.01^a^ 0.71^a^ ± 0.02^b^ 0.70 ± 0.01^b^	0.0132
**P-value Groups**	0.000					
**P-value Days**	0.000					
P-value Groups*Days	0.000					
**EPA** 0 day 14 days 28 days 52 days	0.21 ± 0.00^f^ 0.22 ± 0.01^ef^ 0.22 ± 0.02^ef^ 0.22 ± 0.01^ef^	0.23 ± 0.01^def^ 0.23 ± 0.01^def^ 0.24 ± 0.02^cd^ 0.23 ± 0.00^def^	0.22 ± 0.02^ef^ 0.21 ± 0.01^f^ 0.25 ± 0.01^c^ 0.26 ± 0.01^b^	0.23 ± 0.00^def^ 0.25 ± 0.01^c^ 0.26 ± 0.02^b^ 0.25 ± 0.01^c^	0.23 ± 0.01^def^ 0.27 ± 0.01^a^ 0.27 ± 0.00^a^ 0.26 ± 0.02^b^	0.0139
**P-value Groups**	0.000					
**P-value Days**	0.000					
P-value Groups*Days	0.000					
**n-3PUFA** 0 day 14 days 28 days 52 days	1.53 ± 0.10^g^ 1.52 ± 0.15^g^ 1.52 ± 0.11^g^ 1.50 ± 0.11^g^	1.50 ± 0.12 ^g^ 0.96 ± 0.15^h^ 1.18 ± 0.15^h^ 1.21 ± 0.16^h^	1.50 ± 0.16^g^ 1.92 ± 0.16^f^ 2.30 ± 0.05^e^ 2.20 ± 0.07^e^	1.51 ± 0.15^g^ 2.81 ± 0.15^d^ 3.50 ± 0.07^c^ 3.40 ± 0.12^c^	1.50 ± 0.12^g^ 4.77 ± 0.17^a^ 4.14 ± 0.12^b^ 4.75 ± 0.09^a^	0.1291
**P-value Groups**	0.001					
**P-value Days**	0.011					
P-value Groups*Days	0.001					
**n-6PUFA** 0 day 14 days 28 days 52 days	15.66 ± 0.43 15.66 ± 0.42 16.26 ± 0.31 16.00 ± 0.33	15.65 ± 0.31 16.25 ± 0.31 16.91 ± 0.35 17.48 ± 0.39	15.66 ± 0.37 16.35 ± 0.35 17.49 ± 0.40 17.48 ± 0.41	15.69 ± 0.47 16.35 ± 0.36 18.10 ± 0.32 17.71 ± 0.29	15.63 ± 0.25 20.81 ± 0.28 19.85 ± 0.25 20.71 ± 0.23	0.3212
**P-value Groups**	0.098					
**P-value Days**	0.120					
P-value Groups*Days	0.135					
**n-6/3PUFA** 0 day 14 days 28 days 52 days	10.31 ± 0.19^c^ 10.30 ± 0.17^c^ 10.67 ± 0.24^c^ 10.66 ± 0.22^c^	10.40 ± 0.25^c^ 16.95 ± 0.26^a^ 14.31 ± 0.18^b^ 14.46 ± 0.22^b^	10.43 ± 0.28^c^ 8.28 ± 0.27^d^ 7.61 ± 0.19^e^ 7.91 ± 0.19^e^	10.32 ± 0.21^c^ 5.80 ± 0.19^g^ 5.18 ± 0.23^f^ 5.21 ± 0.25^f^	10.41 ± 0.19^c^ 4.30 ± 0.28^h^ 4.59 ± 0.21^h^ 4.34 ± 0.19^h^	0.2237
**P-value Groups**	0.000					
**P-value Days**	0.000					
P-value Groups*Days	0.000					

Different dosages of Schizochytrium powder (0.25%, 0.50%, 0.75% and 1%), alpha-linolenic acid (ALA), docosahexaenoic acid (DHA), eicosapentaenoic acid (EPA); Omega-3 polyunsaturated fatty acids (n-3 PUFAs); Omega-6 polyunsaturated fatty acids (n-6 PUFAs)

SEM is the standard error of means between groups*days

^a−h^ Different letters represent statistical differences between groups*days within a parameter at *p* ≤ 0.05

## Discussion

The worldwide demand for microalgal foods is increasing, and people are consuming more algae for health and nutritional reasons. Schizochytrium algae reserved 14% DHA in its nutritional content [7]. Biofortification of hen eggs with microalgae in appropriate doses enhances the chances for consumption of omega 3 fatty acids to compensate the omega 6 in today’s diet style so the present study presents a solution to the problem related to this disproportion through daily consumption item, however limited doses have been used in accordance with required ratio. In accordance with the present study, Curabay et al. [20] reported in their investigation that adding 1% and 2% *Spirulina platensis* to poultry diet had a significant favorable impact on egg yolk color but had no influence on other factors, including egg weight, daily feed intake, feed conversion ratio, eggshell thickness. Our findings correspond to previous research on laying hens reported by Moran et al. [21] showed that supplementing microalga with DHA, not only maintained essential egg quality indicators but also increased nutritional profile of eggs, demonstrating the appropriate use of marine microalgae as a poultry feed element. Compared to the control, significant yolk color variations were seen in the first 28 days of administering microalgal diets, and the levels approached a plateau after 52 days, after which they were retained and Bruneel et al. [2] also observed similar trends. Prior to obtaining the plateau, the results changed dramatically due to the longer feeding duration and increasing microalga incorporation levels, which correlated with a drop in brightness and an upsurge in redness of the egg yolk. These results are consistent with the previously investigated supplementation of laying hens with different microalgal species, such as DHA-enriched heterotrophic *Schizochytrium limacinum* [22] and EPA-containing autotrophic Nannochloropsis. In the case of crude fat, content increased along with rising Schizochytrium fortification levels, emphasizing this rise over time, probably due to increased fatty acid consumption and accumulation in the yolk of eggs, especially n-3 fatty acids, as previously reported by Wu et al. [23].

In contrast, Park et al. [24] reported that nutritional supplementation with 1.0% Schizochytrium species positively affected hen production, which is consistent with our findings. As anticipated, adding microalgal DHA to poultry feeds resulted in dose-dependent stimulation of DHA in egg yolks (*P* < 0.05), which was consistent with the preliminary results of Neves et al. [11] using extracted *Nannochloropsis oceanica* microalga. Fatty acid deposition and DHA enrichment in egg yolks depend on feed intake, as well as the duration of fortification. According to our results, 1% microalgal powder reached its plateau point on day 14, after which no further increase was observed likewise in the case of 0.75% and 0.50% microalgal powder saturation point reached on its maximum after 28 days. In the case of 0.25% microalgae, the plateau point was seen on day 52 showed steady enrichment process, as illustrated in Table 4. Hargis et al. [8] and Keegan et al. [25] confirmed comparable outcomes and observed that the minimum period for yolk DHA enrichment to reach a plateau was 4 weeks. Nevertheless, further studies found that maximum n-3 PUFA assimilation was obtained shortly after 2 or 3 weeks of feeding with *Phaeodactylum tricornutum*,* Chlorella fusca* [26] and *Nannochloropsis* [27]. This disparity might be attributed to changes in microalgal species, as well as the hen’s age and strain. Upon hitting the plateaus, DHA levels in the yolks from laying hens provided 1.0% microalga increased nearly 3-fold above control group.

Humans have evolved a biological tolerance to diets with comparatively low amounts of saturated and total fatty acids. The appropriate ratio of n-6 to n-3 unsaturated fatty acids was approximately 1% whereas, a modern European diet’s n-6/n-3 ratio has altered, mostly because of industrialization. Poultry and seafood are principal producers of EPA and DHA, respectively. de Carvalho et al. [28] provided poultry feed containing 0.5–1.75% marine microalga and recorded no significant differences among the dosages, despite of the highest dose of dietary supplementation, which had considerably more EPA compared to the other groups. Treatments that did not vary in the context of yolk EPA concentration had equal amounts of dietary EPA, demonstrating that yolk EPA content paralleled the diet. Normally, no more than 12 mg of EPA is deposited in the whole yolk. It has recently been proven that the transformation of EPA to DHA is somewhat less effective than direct DHA accumulation, highlighting the value of DHA-rich Schizochytrium algae as an alternative source of n-3 FA [3].

Moreover, in present study, fortification of eggs with DHA via microalgae dietary supplementation boosted the content of n-3 PUFA in the yolk in a dose-dependent manner and consequently reduced the proportion n-6 PUFA to n-3 PUFA, which shows improvement in the content of n-3 compared to n-6 PUFA [2, 23]. Increasing n-3 PUFA concentrations and decreasing the n-6-to-n-3 ratio within human diets have a significant impact on lowering the risk of heart disease and boosting neural growth and performance. After 1% microalgae supplementation, the n-6/n-3 ratios decreased from the value in the control yolk (*p* ≤ 0.05) a trend which was consistent with previous data and may correspond to an optimal ratio for human health [29].

## Conclusion

The present study resulted in increased levels of ALA, DHA, and EPA in hen eggs in dose dependent manner and also improved omega 6 to omega 3 ratio after addition of Schizochytrium powder in laying hen’s feed. Especially 1% dose was found to be more efficient and it reached its plateau point after 14 days of feed consumption, which was maintained until the end of the study period (day 52), while other doses took 28 to 52 days to reach the level. Therefore, it can be concluded that 1% level of feed supplementation with Schizochytrium algae powder provides an excellent vegetarian source of omega 3-PUFA to enrich the eggs in a minimum time that is maintained for longer period of time.

## Data Availability

Even though adequate data has been given in the form of tables however, all authors declare that if more data required then the data will be provided on request basis.

## Abbreviations

DHA: Docosahexaenoic acid
ALA: α-linolenic acid
EPA: Eicosapentaenoic acid
PUFAs: Polyunsaturated fatty acids
DPA: Docosapentaenoic acid
EFSA: European Food Safety Authority
FCR: Feed conversion ratio
FAME: Fatty acid methyl esters
LSD: Least significant difference
HU: Haugh unit
DFI: Daily feed intake
n-3 PUFAs: Omega-3 polyunsaturated fatty acids
n-6 PUFAs: Omega-6 polyunsaturated fatty acids

## References

[1] Baker MT, Lu P, Parrella JA, Leggette HR. Consumer acceptance toward functional foods: a scoping review. Int J Environ Res Public Health. 2022;19(3):1217. 10.3390/ijerph19031217. 35162240 10.3390/ijerph19031217 PMC8835010

[2] Bruneel C, Lemahieu C, Fraeye I, Ryckebosch E, Muylaert K, Buyse J, Foubert I. Impact of microalgal feed supplementation on omega-3 fatty acid enrichment of hen eggs. J Funct Foods. 2013;5(2):897–904.

[3] Gładkowski W, Kiełbowicz G, Chojnacka A, Bobak Ł, Spychaj R, Dobrzański Z, Trziszka T, Wawrzeńczyk C. The effect of feed supplementation with dietary sources of n-3 polyunsaturated fatty acids, flaxseed and algae Schizochytrium sp., on their incorporation into lipid fractions of Japanese quail eggs. Int J Food Sci Technol. 2014;49(8):1876–85.

[4] Kralik Z, Kralik G, Grčević M, Hanžek D, Margeta P. Microalgae Schizochytrium limacinum as an alternative to fish oil in enriching table eggs with n-3 polyunsaturated fatty acids. J Sci Food Agric. 2020;100(2):587–94. 31591720 10.1002/jsfa.10052

[5] Moran C, Morlacchini M, Keegan J, Fusconi G. Increasing the omega-3 content of hen’s eggs through dietary supplementation with Aurantiochytrium Limacinum microalgae: effect of inclusion rate on the temporal pattern of docosahexaenoic acid enrichment, efficiency of transfer, and egg characteristics. J Appl Poult Res. 2019;28(2):329–38.

[6] DiNicolantonio JJ, O’Keefe J. The importance of maintaining a low omega-6/omega-3 ratio for reducing the risk of autoimmune diseases, asthma, and allergies. Mo Med. 2021;118(5):453–9. 34658440 PMC8504498

[7] Kaewsutas M, Sarikaphuti A, Nararatwanchai T, Sittiprapaporn P, Patchanee P. The effects of dietary microalgae (Schizochytrium spp.) and fish oil in layers on docosahexaenoic acid omega-3 enrichment of the eggs. J Appl Anim Nutr. 2016;4:e7. 10.1017/jan.2016.4.

[8] Hargis P, Van Elswyk M, Hargis B. Dietary modification of yolk lipid with menhaden oil. Poult Sci. 1991;70(4):874–83. 1908579 10.3382/ps.0700874

[9] Lemahieu C, Bruneel C, Termote-Verhalle R, Muylaert K, Foubert I, Buyse J. Dynamics of omega‐3 long chain polyunsaturated fatty acid incorporation in egg yolk by autotrophic microalgal supplementation. Eur J Lipid Sci Technol. 2015;117(9):1391–7.

[10] Carrillo S, Ríos VH, Calvo C, Carranco ME, Casas M, Pérez-Gil F. N-3 fatty acid content in eggs laid by hens fed with marine algae and sardine oil and stored at different times and temperatures. J Appl Phycol. 2012;24:593–9. 10.1007/s10811-011-9777-x.

[11] Neves M, Ferreira A, Antunes M, Laranjeira Silva J, Mendes S, Gil MM, Tecelão C. Nannochloropsis Oceanica as a sustainable source of n-3 polyunsaturated fatty acids for enrichment of hen eggs. Appl Sci. 2021;11(18):8747. 10.3390/app11188747.

[12] Adrizal A, Yusrizal Y, Fakhri S, Haris W, Ali E, Angel C. Feeding native laying hens diets containing palm kernel meal with or without enzyme supplementations: feed conversion ratio and egg production. J Appl Poult Res. 2011;20(1):40–9.

[13] Chen X, Li X, Guo Y, Li W, Song J, Xu G, Yang N, Zheng J. Impact of cuticle quality and eggshell thickness on egg antibacterial efficiency. Poult Sci. 2019;98(2):940–8. 30137530 10.3382/ps/pey369

[14] Silversides F, Budgell K. The relationships among measures of egg albumen height, pH, and whipping volume. Poult Sci. 2004;83(10):1619–23. 15510543 10.1093/ps/83.10.1619

[15] Lynch JM, Barbano DM. Kjeldahl nitrogen analysis as a reference method for protein determination in dairy products. J AOAC Int. 1999;82(6):1389–98. 10589493

[16] Carrillo S, López E, Casas M, Avila E, Castillo R, Carranco M, Calvo C, Pérez-Gil F. Potential use of seaweeds in the laying hen ration to improve the quality of n-3 fatty acid enriched eggs. In *Nineteenth International Seaweed Symposium: Proceedings of the 19th International Seaweed Symposium, held in Kobe, Japan, 26–31 March*, 2009; Springer: pp 271–278.

[17] Thiex N, Novotny L, Crawford A. Determination of ash in animal feed: AOAC official method 942.05 revisited. J AOAC Int. 2012;95(5):1392–7. 23175971 10.5740/jaoacint.12-129

[18] Feng J, Long S, Zhang H-j, Wu S-g, Qi G-h, Wang J. Comparative effects of dietary microalgae oil and fish oil on fatty acid composition and sensory quality of table eggs. Poult Sci. 2020;99(3):1734–43. 32115040 10.1016/j.psj.2019.11.005 PMC7587657

[19] Dillon GP, Yiannikouris A, Brandl W, Cardinall C, Yuan W, Moran CA. Analytical method assessment for the determination of DHA and fatty acids present in unextracted Aurantiochytrium limacinum biomass. Food Nutr Sci. 2019;10(04):469.

[20] Curabay B, Sevim B, Cufadar Y, Ayasan T. Effects of adding spirulina platensis to laying hen rations on performance, egg quality, and some blood parameters. J Hellenic Vet Med Soc. 2021;72(2):2945–52.

[21] Moran C, Morlacchini M, Keegan J, Rutz F, Fusconi G. Docosahexaenoic acid enrichment of layer hen tissues and eggs through dietary supplementation with heterotrophically grown Aurantiochytrium Limacinum. J Appl Poult Res. 2020;29(1):152–61.

[22] Ao T, Macalintal L, Paul M, Pescatore A, Cantor A, Ford M, Timmons B, Dawson KA. Effects of supplementing microalgae in laying hen diets on productive performance, fatty-acid profile, and oxidative stability of eggs. J Appl Poult Res. 2015;24(3):394–400.

[23] Wu Y, Li L, Wen Z, Yan H, Yang P, Tang J, Xie M, Hou S. Dual functions of eicosapentaenoic acid-rich microalgae: enrichment of yolk with n-3 polyunsaturated fatty acids and partial replacement for soybean meal in diet of laying hens. Poult Sci. 2019;98(1):350–7. 30203026 10.3382/ps/pey372

[24] Park J, Upadhaya S, Kim I. Effect of dietary marine microalgae (Schizochytrium) powder on egg production, blood lipid profiles, egg quality, and fatty acid composition of egg yolk in layers. Asian-Australas J Anim Sci. 2015;28(3):391. 10.5713/ajas.14.0463. 25656210 10.5713/ajas.14.0463 PMC4341084

[25] Keegan J, Currie D, Knox A, Moran C. Heterotrophic aurantiochytrium sp. supplementation to layer diets sustainably increases the omega-3 concentration of eggs. Br Poult Sci. 2019;60(5):570–8. 31124696 10.1080/00071668.2019.1622079

[26] Lemahieu C, Bruneel C, Ryckebosch E, Muylaert K, Buyse J, Foubert I. Impact of different omega-3 polyunsaturated fatty acid (n-3 PUFA) sources (flaxseed, Isochrysis galbana, fish oil and DHA Gold) on n-3 LC-PUFA enrichment (efficiency) in the egg yolk. J Funct Foods. 2015;19:821–7.

[27] Kim J, Barcus M, Magnuson A, Tao L, Lei XG. Supplemental defatted microalgae affects egg and tissue fatty acid composition differently in laying hens fed diets containing corn and flaxseed oil. J Appl Poult Res. 2016;25(4):528–38.

[28] de Carvalho PR, Pita MCG, Neto EP, De Mendonça C. Efficiency of PUFAs incorporation from marine sources in yolk egg’s laying hens. Int J Poult Sci. 2009;8(6):603–14.

[29] Erkkilä A, de Mello VD, Risérus U, Laaksonen DE. Dietary fatty acids and cardiovascular disease: an epidemiological approach. Prog Lipid Res. 2008;47(3):172–87. 18328267 10.1016/j.plipres.2008.01.004

